# The association between bone age advancement and insulin in prepubertal children with obesity

**DOI:** 10.1186/1687-9856-2015-S1-P71

**Published:** 2015-04-28

**Authors:** Hae Sang Lee, Hwal Rim Jeong, Eunbyul Kwon, Jin Soon Hwang

**Affiliations:** 1 Department of Pediatrics, Ajou University School of Medicine, Ajou University Hospital, Suwon, Korea

## Aims

Obesity is associated with bone age (BA) advancement of unclear etiology. In animal study, insulin may directly modulate skeletal growth. Our objective was to investigate the association with BA maturation and insulin levels in children with overweight and obesity.

## Methods

In this cross-sectional study of 103 prepubertal children, anthropometric data and hormonal values during oral glucose tolerance test were measured. Subjects were divided into two groups by the difference between BA and chronological age (CA) (noted as BA-CA).

## Results

The study population included 49 (47.6%) males and 54 (52.4%) females with a mean age of 7.6 ± 1.6 years. The advanced bone age group defined as BA-CA>1 year (n=53) had significantly higher HOMA-IR, fasting insulin levels and lower quantitative insulin sensitivity check index (QUICKI). Also, BA-CA was significantly correlated with fasting insulin (r=0.315, P<0.001), HOMA-IR (r=0.288, P<0.001), and QUICKI (r=-0.353, P<0.001). In multiple regression analysis, fasting insulin was identified as significant independent predictors for BA-CA.

## Conclusion

Skeletal age is more advanced in overweight and obese children with hyperinsulinemia and insulin resistance. These findings suggest that insulin may modulate skeletal growth.

